# Drivers of the relative richness of naturalized and invasive plant species on Earth

**DOI:** 10.1093/aobpla/plz051

**Published:** 2019-09-04

**Authors:** Franz Essl, Wayne Dawson, Holger Kreft, Jan Pergl, Petr Pyšek, Mark Van Kleunen, Patrick Weigelt, Thomas Mang, Stefan Dullinger, Bernd Lenzner, Dietmar Moser, Noëlie Maurel, Hanno Seebens, Anke Stein, Ewald Weber, Cyrille Chatelain, Inderjit, Piero Genovesi, John Kartesz, Olga Morozova, Misako Nishino, Pauline M Nowak, Shyama Pagad, Wen-Sheng Shu, Marten Winter

**Affiliations:** 1 Division of Conservation, Landscape and Vegetation Ecology, Department of Botany and Biodiversity Research, University of Vienna, Vienna, Austria; 2 Department of Biosciences, Durham University, Durham, UK; 3 Biodiversity, Macroecology and Biogeography, University of Goettingen, Göttingen, Germany; 4 The Czech Academy of Sciences, Institute of Botany, Department of Invasion Ecology, Průhonice, Czech Republic; 5 Department of Ecology, Faculty of Science, Charles University, Prague, Czech Republic; 6 Centre for Invasion Biology, Department of Botany and Zoology, Stellenbosch University, Matieland, South Africa; 7 Ecology, University of Konstanz, Konstanz, Germany; 8 Zhejiang Provincial Key Laboratory of Plant Evolutionary Ecology and Conservation, Taizhou University, Taizhou, China; 9 Senckenberg Biodiversity and Climate Research Centre (BiK-F), Frankfurt am Main, Germany; 10 Institute of Biochemistry and Biology, University of Potsdam, Potsdam, Germany; 11 Conservatoire et Jardin Botaniques de la Ville de Genève, Genève, Switzerland; 12 Department of Environmental Studies and Centre for Environmental Management of Degraded Ecosystems, University of Delhi, Delhi, India; 13 Institute for Environmental Protection and Research (ISPRA), Rome, Italy; 14 Chair IUCN Species Survival Commission’s Invasive Species Specialist Group (ISSG), Rome, Italy; 15 Biota of North America Program (BONAP), Chapel Hill, NC, USA; 16 Institute of Geography RAS, Staromonetny, Moscow, Russia; 17 Department of Geography, University Marburg, Deutschhausstraße, Marburg, Germany; 18 IUCN Species Survival Commission’s Invasive Species Specialist Group (ISSG), University of Auckland, Auckland, New Zealand; 19 State Key Laboratory of Biocontrol and Guangdong Key Laboratory of Plant Resources, College of Ecology and Evolution, Sun Yat-sen University, Guangzhou, China; 20 German Centre for Integrative Biodiversity Research (iDiv) Halle-Jena-Leipzig, Leipzig, Germany

**Keywords:** Alien species richness, biogeography, invasion stages, islands, pressures, vascular plants

## Abstract

Biological invasions are a defining feature of the Anthropocene, but the factors that determine the spatially uneven distribution of alien plant species are still poorly understood. Here, we present the first global analysis of the effects of biogeographic factors, the physical environment and socio-economy on the richness of naturalized and invasive alien plants. We used generalized linear mixed-effects models and variation partitioning to disentangle the relative importance of individual factors, and, more broadly, of biogeography, physical environment and socio-economy. As measures of the magnitude of permanent anthropogenic additions to the regional species pool and of species with negative environmental impacts, we calculated the relative richness of naturalized (= RRN) and invasive (= RRI) alien plant species numbers adjusted for the number of native species in 838 terrestrial regions. Socio-economic factors (per-capita gross domestic product (GDP), population density, proportion of agricultural land) were more important in explaining RRI (~50 % of the explained variation) than RRN (~40 %). Warm-temperate and (sub)tropical regions have higher RRN than tropical or cooler regions. We found that socio-economic pressures are more relevant for invasive than for naturalized species richness. The expectation that the southern hemisphere is more invaded than the northern hemisphere was confirmed only for RRN on islands, but not for mainland regions nor for RRI. On average, islands have ~6-fold RRN, and >3-fold RRI compared to mainland regions. Eighty-two islands (=26 % of all islands) harbour more naturalized alien than native plants. Our findings challenge the widely held expectation that socio-economic pressures are more relevant for plant naturalization than for invasive plants. To meet international biodiversity targets and halt the detrimental consequences of plant invasions, it is essential to disrupt the connection between socio-economic development and plant invasions by improving pathway management, early detection and rapid response.

## Teaser

The human-mediated introduction of species outside their native range is considered to be one of the greatest threats to biodiversity, yet a comprehensive analysis of the drivers of global invasions is currently lacking. Here, we use a novel database of naturalized and invasive plant species richness to analyse the effects of biogeographical, physical environment and socio-economic factors on the numbers of naturalized and invasive plant species relative to native species in 838 terrestrial regions. We demonstrate that the numbers of naturalized and invasive plant species relative to native species richness is highest on (sub)tropical islands, and that socio-economic factors foster invasive spread after naturalization. To meet international biodiversity targets and to halt the detrimental consequences of plant invasions, it is essential to disrupt the connection between socio-economic development and rising levels of plant invasions by improving pathway management, early detection and rapid response.

## Introduction

Human-mediated dispersal of species into new regions has become a key feature of the Anthropocene (Waters *et al.* 2016), redefining biogeographical realms (Capinha *et al.* 2015) and homogenizing the worlds’ biota (Winter *et al.* 2010; La Sorte *et al.* 2014). So far, ~4 % of all extant vascular plant species (van Kleunen *et al.* 2015), and similar proportions of birds and mammals (Blackburn *et al.* 2015), have established wild populations beyond their native range, i.e. are naturalized (Blackburn *et al.* 2011). Also the number of naturalized species that have become invasive, i.e. that spread widely (Blackburn *et al.* 2011) and cause deleterious impacts on the environment and human societies (Lambertini *et al.* 2011; Simberloff *et al.* 2013), is substantial (McGeoch *et al.* 2010; Vilà *et al.* 2011) and rapidly increasing (Catford *et al.* 2009; Hulme *et al.* 2013). The numbers of naturalized plant species, however, vary among regions across the globe (Pyšek *et al.* 2017).

A range of hypotheses have been proposed to explain the processes shaping the geographic patterns of alien species distributions (Catford *et al.* 2009). It has been argued that (i) islands are more susceptible to biological invasions compared to mainland regions (Denslow *et al.* 2009; van Kleunen *et al.* 2015; Dawson *et al.* 2017; Moser *et al.* 2018), (ii) regions in the southern hemisphere are more invaded than in the northern hemisphere (Richardson and Pyšek 2012; van Kleunen *et al.* 2015), (iii) New World regions that have been exposed to massive European settlement have received more alien species than Old World regions (di Castri 1989, but see van Kleunen *et al.* 2015), (iv) the extent of biological invasions differs between regions in relation to current and historical human impact (Pyšek *et al.* 2010; Essl *et al.* 2011) and (v) that socio-economic pressures are more relevant for naturalized species richness than for invasive species richness (Williamson 2006). Although much research has been devoted to testing the validity of these hypotheses and improved insights are highly needed for invasive alien species policy and management, a comprehensive analysis of the drivers mediating richness of naturalized and invasive plants at global extent is still missing.

Biological invasions are interpreted as a sequence of stages separated by barriers that a species has to overcome before becoming a successful invader (Blackburn *et al.* 2011), and regional studies have shown that the underlying drivers can substantially differ among invasion stages (Dawson *et al.* 2009, 2013; Richardson and Pyšek 2012). For instance, while socio-economic drivers underlying colonization effort are deemed crucial for introduction and naturalization (Williamson 2006), invasiveness is assumed to depend more on species’ traits (Pyšek *et al.* 2009) as well as the interaction of alien species with native biota and the physical environment of the recipient region.

Recently, Dawson *et al.* (2017) presented a cross-taxonomic study (based on eight taxonomic groups, including vascular plants) on global patterns and underlying drivers of naturalized alien species richness. In their study, they identified hotspots of naturalized plants and animals, found that these hotspots are primarily located on islands and coastal regions, and that socio-economic variables were particularly important for naturalized species richness. However, Dawson *et al.* (2017) differ in key attributes from this study: they focused on absolute species numbers and on a single invasion stage (i.e. richness of naturalized species), and their analyses of underlying drivers were based on a smaller set of explanatory variables.

Here, we advance previous studies and present the most comprehensive global analysis, to our knowledge, of the patterns and drivers of alien plant species richness. We focus on two key stages in the spread of alien plants, i.e. naturalized plants (= permanently established alien species) and invasive plants (= alien species that spread widely and cause negative impacts on the environment; CBD 2000), because the main drivers of these stages might differ. We calculated the relative richness of naturalized and invasive plants as the numbers of naturalized and invasive species relative to the number of native species in a region. While the relative richness of naturalized species (RNN) is quantifying the magnitude of permanent anthropogenic additions to the regional species pool, the relative richness of invasive species (RRI) considers alien species that cause negative environmental impacts. By relating alien to native species richness, the metrics used illustrate how the contribution of alien species to entire regional species pools varies across the globe instead of just demonstrating patterns of alien species numbers. Native plant species richness on a global scale is strongly driven by energy availability (temperature, amount and timing of precipitation) and environmental heterogeneity (Hawkins *et al.* 2003), and it is further moderated by historical legacy of climatic and evolutionary factors (Kreft and Jetz 2007). This makes native species richness a better proxy for assessing the regional variation in plant invasions compared to other variables such as the widely used region size, as area does not account for the above-mentioned factors. We acknowledge that alternative metrics such as absolute numbers of alien species (Pyšek *et al.* 2010; Essl *et al.* 2011; Dawson *et al.* 2017; Moser *et al.* 2018), or the proportion of naturalized species that have become invasive (Dawson *et al.* 2009) have been used for testing hypotheses of biological invasions. Both absolute and relative richness of alien plant species deliver important and complementary insights into the drivers of invasions. The relative metrics we use here are particularly suited to shedding light on the factors that drive the magnitude of changes brought about by biological invasions.

## Materials and Methods

### Data

#### Geographic coverage and regions selection

**Figure 1.**
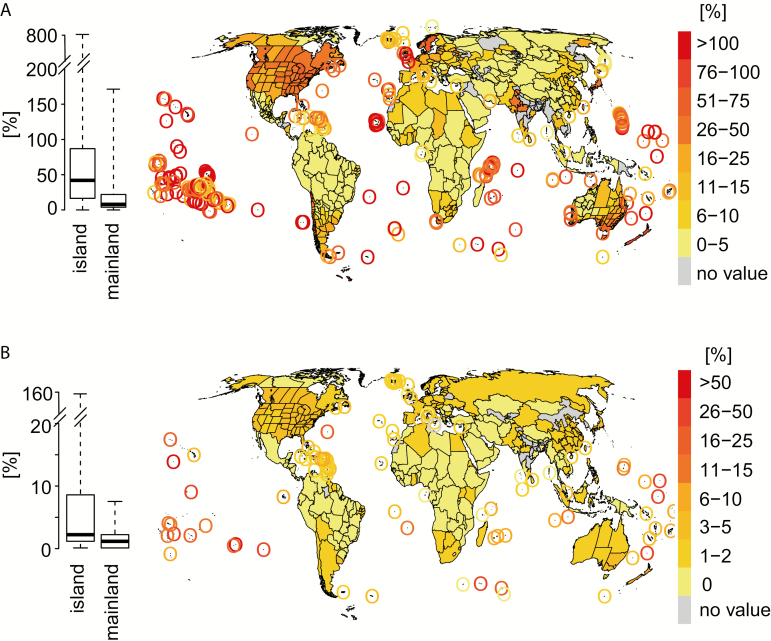
The global biogeography of alien vascular plant species. Shown are the relative richness of naturalized (RRN) (A) (*n* = 761 regions) and (B) of invasive (RRI) (*n* = 359 regions) plants, i.e. ratios of naturalized and invasive vascular plant species in relation to native species, respectively. Boxplots show the relative richness of naturalized and invasive plants for island and mainland regions. Differences between island and mainland regions were assessed by Mann–Whitney *U* tests, and are highly significant in both cases (*P* < 0.001).

The Global Naturalized Alien Flora (GloNAF) database (van Kleunen *et al.* 2015, 2019; Pyšek *et al.* 2017) aims to cover the total ice-free terrestrial surface area of the globe, divided into countries and—in particular for large countries and whenever data allowed—subnational regions (e.g. provinces, federal states, islands; van Kleunen *et al.* 2015; Pyšek *et al.* 2017). In total, GloNAF includes 1082 regions with a varying degree of completeness of information. In many cases, regions with different degrees of spatial resolution overlap or are nested (e.g. US federal states nested in the USA). Therefore, we have selected a subset of 838 spatially non-overlapping regions (composed of 495 continental regions such as countries or federal states, and 343 islands) based on data availability and the criterion of highest available spatial resolution. For 761 regions there is information on naturalized plant species richness, and for 359 regions on invasive plant species richness (Fig. 1). We extended the data with information on numbers of native species for all regions. While the number of regions containing data on invasive species is substantially smaller because of a lack of data mostly for smaller subnational regions and islands, global data coverage remains high (c. 90 % of global terrestrial area) (cf. **Supporting Information—Fig. S1C**).

#### Native, naturalized and invasive alien plant species data compilation

The GloNAF database includes inventories of naturalized alien plant species (also including infraspecific taxa such as subspecies and hybrid taxa) for 761 regions around the globe **[see****Supporting Information—Table S3****]**. We consider alien species that have established self-sustaining populations without direct human intervention to be naturalized following Richardson *et al.* (2000), Pyšek *et al.* (2004), Blackburn *et al.* (2011) and Essl *et al.* (2018). The data sources include naturalized alien plant compendia, national and subnational lists of naturalized alien plant species published in scientific journals, as books or on the internet, as well as compendia of national or subnational floras with information on which species occur in the wild but are not native (van Kleunen *et al.* 2019). Our database also includes unpublished inventories of naturalized alien species that were specifically compiled for the GloNAF database (e.g. for the provinces of China and the states of India) **[see****Supporting Information—Table S3****]**.

Invasive alien species are a subset of naturalized alien species which often spread widely from the point of introduction (Richardson *et al.* 2000; Blackburn *et al.* 2011) and cause negative impacts on the environment (Blackburn *et al.* 2014). Somewhat different approaches to define invasive alien species exist. Here, we followed the most widely accepted approach in environmental policy, the standard definition of the CBD (2000): ‘…an alien species whose introduction and/or spread threatens biological diversity’, in which the term ‘invasive’ in effect consists of the subset of naturalized alien species which exert negative impacts on the environment. This focus on negative impacts differs somewhat from the definition of Richardson *et al.* (2000), which focuses on the ability to spread and is widely used in invasion ecology: alien species that sustain self-replacing populations over several life cycles, produce reproductive offspring, often in very large numbers at considerable distances from the parent population and/or site of introduction, and which have the potential to spread over long distances. Both definitions are nevertheless compatible and even if the criteria may slightly differ in individual cases, the resulting lists of invasive species are likely similar in global terms.

To avoid the influence of different interpretations of the term invasive and to ensure a geographically balanced sampling, we based our consensus list of invasive alien plant species on three global data sources which contain standardized information on invasiveness: (i) the CABI Invasive Species Compendium (http://www.cabi.org/isc/), which contains 672 plant data sheets with information on invasiveness in national and subnational regions, (ii) the ISSG Global Invasive Species Database (http://www.iucngisd.org/gisd), which contains information on invasiveness of 2530 plant species, and (iii) the invasive plant species database by Weber (2017) with information on 107 invasive plant species. By using these global data sources, we are convinced that remaining uncertainties (e.g. different interpretations of invasiveness) are moderate. We extracted invasive plant species from these three data sources, standardized their scientific names (see below) and then built a consensus list for each GloNAF region including the invasive species recorded in these data sources. This approach yielded data of invasive alien plant species numbers for 359 regions of the world.

We aimed at including the most comprehensive and most recent regional inventories. Indeed, >95 % of the data sources are from the last two decades. Moreover, since some of the original source lists included alien species that are cultivated only or have non-persistent populations in the wild, we excluded those species whenever such information was provided, or contacted experts of the regional floras to filter out species of doubtful naturalization status. Furthermore, for European countries that differentiated between archaeophytes (alien species that arrived until the year 1492) and neophytes (species that arrived after the year 1492), we kept only the latter, because the alien status of some species classified as archaeophytes is disputed; moreover, this classification is not available for most other regions of the world, which would prevent us from achieving a standardized assessment of naturalized and invasive alien species numbers.

To standardize scientific names, each naturalized and invasive plant inventory was compared to The Plant List (TPL 2014, version 1.1; http://www.theplantlist.org/; see van Kleunen *et al.* 2015 for further details), the most comprehensive working list of all plant species (Kalwij 2012). This taxonomic standardization was done with the help of the R package *Taxonstand* (Cayuela and Oksanen 2014). For each species, we kept the name accepted by The Plant List. Species that were not found in The Plant List, also not after accounting for spelling differences, were kept in the database using the names as used in the source data. In total, the database includes 13 168 species of which 13 033 are recognized by The Plant List (12 498 as accepted and 535 as unresolved names). The remaining 135 species could not be identified as they do not occur in The Plant List, and among those 11 are ornamental cultivars of unknown origins.

Native plant species numbers were also extracted from national checklists and standard floras. We preferably used continent-wide standard floras such as the BONAP database for North America (http://www.bonap.org/). For islands, we extracted data on native plant species numbers from the GIFT database (http://gift.uni-goettingen.de/) compiled by H. Kreft and P. Weigelt (Weigelt *et al.* 2015, 2016). The few remaining gaps were closed by consulting national experts.

#### Biogeographic, climatic, environmental and socio-economic variables

For each GloNAF region, we collected a range of variables which are assumed to relate to current RRN (= number of naturalized species relative to native species number) and RRI (= number of invasive species relative to native species number). Each GloNAF region was assigned to one of nine continents of the TDWG continental scheme (further referred to as TDWG continents; Brummit 2001). We calculated the area of each region while considering only the ice sheet-free areas of each region, ranging from 0.03 to 4 331 903 km^2^ (median: 28 836 km^2^). In addition, each GloNAF region was uniquely classified as belonging to a continental landmass (= mainland) or island, assigned to a hemisphere, Old/New World and the predominant broad climatic regime (zonobiome) to which the dominant vegetation type is closely associated (according to the Köppen-Geiger climate classification system; Kottek *et al.* 2006; Peel *et al.* 2007). Spatial heterogeneity positively influences the number of niches available, which in turn may be colonized by alien plants. We thus used topographic heterogeneity of the region, calculated as the standard deviation of the mean elevation of each grid cell (size: 30 × 30 geographic seconds, corresponding to c. 1 × 1 km at the equator; taken from http://www2.jpl.nasa.gov/srtm/) belonging to the respective region, as a proxy for environmental heterogeneity. Variation in elevation is closely related to variation in climate, geology, geomorphology and land use, and therefore it is a useful measure of a region’s heterogeneity (Kerr and Packer 1997).

Isolation (Moser *et al.* 2018) and the length of human presence have been suggested to be important predictors for plant invasions on islands. Thus, for island regions, we additionally used recently published measures of their isolation (distance to the nearest mainland and landmass; Weigelt and Kreft 2013), and compiled both pre-historic (first colonization by humans) and modern (first settlement by Europeans or descendants of Europeans) settlement dates **[see****Supporting Information—Table S3****]**. Other potentially relevant variables characterizing islands such as being an oceanic or continental island, or if islands have historically been connected to the mainland (Weigelt *et al.* 2016) are closely correlated with isolation; thus, we did not consider these in the analyses.

We selected indicators of human socio-economic activities that are known to affect alien species invasions: land use, i.e. the proportion of agricultural land (Chytrý *et al.* 2008b), and human population density (Pyšek *et al.* 2010; Essl *et al.* 2011) per region. We calculated the proportion of agricultural land and population density using data from the HYDE database (Klein Goldewijk *et al.* 2011). This is a spatially (5ʹ longitude/latitude grid) and temporally high-resolution data set representing 12 000 years (10 000 BC to 2000 AD) of human-induced global land-use change. Data on current climatic conditions (mean annual temperature, mean precipitation) were extracted from the WorldClim database (www.worldclim.org; Hijmans *et al.* 2005). These data were used to calculate GloNAF-region means by averaging over all grid cells for each region. All these variables reflect biogeographic, environmental and socio-economic factors that may modulate the RRN and RRI across the globe.

### Statistical analysis

#### Generalized linear mixed-effects models

We analysed the numbers of naturalized and invasive species dependent on region variables by means of generalized linear mixed-effects models (GLMMs; Faraway 2006). We fitted two different GLMMs with richness of naturalized (Table 1A) and of invasive per native species (Table 1B) as response variables. To account for differences in the number of native species per regions, we used GLMMs with absolute numbers of alien species as Poisson-distributed response variables and the natural logarithm of native species numbers as an offset variable. We used the canonical log-link function. In this setup, the modelled response was effectively a Poisson-rate (rate of alien species per native species). Consequently, for each predictor the GLMMs assessed whether the particular predictor resulted in more (positive coefficient) or fewer (negative coefficient) alien species relative to native species. The number of regions per model varied due to data availability of naturalized and invasive species numbers.

**Table 1.** Generalized linear mixed-effects models of the factors explaining the richness of naturalized (RRN) (A) and invasive (RRI) (B) plants relative to native species of 761 and 359 regions worldwide, respectively. Generalized linear mixed-effects models use a Poisson-rate as response and a total of 14 predictor variables (see Materials and Methods). Note that data on human colonization were only available for islands. Predictors were assessed for significantly different effects on mainland vs. island regions by means of interactions with this binary factor, and subjecting these interactions to a backward model search based on lower AIC; for the retained terms each of the two separate coefficients for mainland and island regions states the predictor effect at an absolute scale. Random-effect intercept terms with sovereign state, TDWG continent and zonobiome as (orthogonal) grouping factors acknowledge for political/socio-economic, biogeographic, spatial and climatic correlations among regions, with an additional observation-level random-effect term accounting for Poisson-distribution overdispersion. Numerical predictor variables were standardized. Estimated standard deviations of random effects: (A) sovereign states: 0.57, TDWG continent: 0.35, zonobiome: 0.20, observation level: 0.62; (B) sovereign states: 0.70, TDWG continent: 0.37, zonobiome: 0.27, observation level: 0.44.

A				
Predictor	Mainland vs. island regions	Coefficient	SE	*P*-value
B				
Predictor	Mainland vs. island regions	Coefficient	SE	*P*-value
Intercept—mainland regions	mainl	−2.67	0.23	<0.001
Intercept—island regions	isl	−0.84	0.27	<0.01
Biogeographic variables				
Southern hemisphere	mainl	−0.16	0.17	0.35
	isl	0.33	0.16	0.05
New World	mainl and isl	0.25	0.24	0.30
Distance to nearest mainland	isl	0.62	0.13	<0.001
Distance to nearest landmass	isl	0.05	0.06	0.35
Physical environment variables				
Topographic heterogeneity	mainl	−0.18	0.06	<0.01
	isl	0.19	0.07	<0.01
Average annual temperature (linear term)	mainl and isl	−0.39	0.08	<0.001
Average annual temperature (quadratic term)	mainl and isl	−0.12	0.04	<0.01
Area	mainl and isl	−0.37	0.10	<0.001
Humidity (aridity index)	mainl	−0.07	0.05	0.19
	isl	−0.63	0.08	<0.001
Socio-economic variables				
Proportion of agricultural land	mainl	0.29	0.06	<0.001
	isl	0.00	0.09	0.99
Population density	mainl	0.25	0.06	<0.001
	isl	0.09	0.07	0.22
Per-capita GDP	mainl and isl	0.27	0.06	<0.001
Year of pre-historic settlement	isl	0.07	0.07	0.27
Year of modern colonization	isl	0.07	0.07	0.31
				
Intercept—mainland regions	mainl	−5.12	0.24	<0.001
Intercept—island regions	isl	−4.13	0.31	<0.001
Biogeographic variables				
Southern hemisphere	mainl and isl	−0.01	0.17	0.95
New World	mainl and isl	0.36	0.30	0.24
Distance to nearest mainland	isl	0.73	0.18	<0.001
Distance to nearest landmass	isl	−0.04	0.14	0.80
Physical environment variables				
Topographic heterogeneity	mainl and isl	−0.14	0.06	0.01
Average annual temperature (linear term)	mainl	−0.19	0.11	0.09
	isl	0.25	0.16	0.12
Average annual temperature (quadratic term)	mainl	−0.11	0.06	0.07
	isl	0.17	0.11	0.13
Humidity (aridity index)	mainl and isl	−0.06	0.07	0.35
Area	mainl and isl	−0.09	0.09	0.34
Socio-economic variables				
Proportion of agricultural land	mainl	0.35	0.06	<0.001
	isl	−0.07	0.09	0.43
Population density	mainl and isl	0.03	0.07	0.71
Per-capita GDP	mainl and isl	0.37	0.07	<0.001
Year of pre-historic settlement	isl	−0.03	0.13	0.83
Year of modern colonization	isl	0.26	0.17	0.12

All models used the region variables as fixed-effect predictors of alien species numbers **[see****Supporting Information—Table S1****]**. As biogeographic variables, we used mainland vs. island region, region area, northern vs. southern hemisphere location, and Old vs. New World location. For islands, we additionally used the distance to the nearest continental landmass and the distance to the nearest landmass (including islands). As physical environment variables, we used topographic heterogeneity, average annual temperature and humidity (measured by the aridity index that is calculated as annual precipitation divided by potential evapotranspiration, and was taken from the Global Aridity and PET database, http://www.cgiar-csi.org; Zomer *et al.* 2008). As socio-economic variables, we used human population density, per-capita gross domestic product (GDP), and the proportion of agricultural area. For island regions, we further used the year of first pre-historic settlement and the year of first modern colonization (for yet uninhabited islands we used 2100 as replacement value). As data screening indicated a potentially pronounced curvi-linear relationship between the response variables and average annual temperature, this predictor entered all models with a linear and quadratic term; for all other predictors only linear terms were used.

To assess predictor variables for different effects on mainland vs. island regions, for all predictors except region area and those available only for island regions, initially separate coefficients were used for mainland vs. island regions by means of interaction terms with this binary factor. However, to reduce models’ complexity and improve numerical stability these interactions were then subjected to a stepwise backward model selection and removed if the simplified model had a lower Akaike Information Criterion (AIC) value (Akaike 1974).

To account for correlative effects of political/socio-economic, biogeographic, and environmental factors and spatial autocorrelation due to selection of regions and their distribution on Earth, the GLMMs included (normally distributed) random-effect intercepts with sovereign states (i.e. all regions belonging to a country, but separated for each TDWG continent in the case of overseas territories), TDWG continent and zonobiome as orthogonal grouping variables. We further added an observation-level random effect to handle possible Poisson-distribution overdispersion (Bolker *et al.* 2009; Harrison 2014). To improve the symmetry of predictor variables, linearity with the modelled response variables and to stabilize variances, numerical predictors were subjected to appropriate transformations (power or log) if so indicated, and furthermore standardized (subtraction of sample mean and division by sample standard deviation). Multicollinearity among predictors was tested by means of eigenvalue ratios and was found to be unproblematic (highest condition number of any GLMM: 9.8). All analyses were conducted using R (R Development Core Team 2015), with the GLMMs fitted by the function *glmer* from the package *lme4* (Bates *et al.* 2014).

#### Relative importance and variation partitioning analyses

Analyses of relative predictor importance and variation partitioning required plain linear models. We therefore worked on the linear predictor scale of the GLMMs by using log-transformed responses of species ratios (naturalized or invasive species numbers with native species numbers as offset variable) and absolute species numbers. In a first step, we fitted linear mixed-effects models (LMMs) using the same fixed- and random-effects structure as for each corresponding GLMM (except for the observation-level random effect which is not applicable to normal-distribution models), and subtracted the linear predictor contribution of all random effects from response values to yield a response adjusted for the impact of these random effects. We then fitted linear models using all fixed-effects variables (including the higher-order terms) of the GLMMs as predictors.

For relative importance analyses, we grouped all base and higher-order terms of a predictor and performed an *R*^2^ partitioning by averaging over orders (Lindeman *et al.* 1980) using the function *calc.relimp* from the R-package *relaimpo* (Grömping 2006). For variation partitioning based on adjusted *R*^2^, we defined three major groups of variables (again including all higher-order terms of predictors), comprising (i) all biogeographic variables; (ii) all physical environment variables; and (iii) all socio-economic variables. Mainland vs. island region and region area were used as core predictors included in all predictor subsets fitted for these analyses and ensured that interaction terms were supplemented by their respective base *terms.*

## Results

### The relative richness of naturalized and invasive plants worldwide

Most naturalized plant species are found in temperate to subtropical mainland regions **[see****Supporting Information—Fig. S1B****]**, whereas the absolute numbers of native plant species peak in tropical mainland regions **[see****Supporting Information—Fig. S1A****]**. The highest absolute numbers of invasive plant species are also reported for temperate and subtropical regions, but show a stronger decline towards tropical regions than naturalized species **[see****Supporting Information—Fig. S1C****]**.

After accounting for native species richness, RRN and RRI show a markedly different pattern across the globe. In total, 82 islands (i.e. 26 % of all islands in the data set) have accumulated more naturalized than native species (Fig. 1A; median: 48.1 naturalized species per 100 native species). Highest RRN values are found on islands in the subtropics and tropics. In contrast, RRN was on average much lower in mainland regions (median: 7.8 naturalized species per 100 native species). The only mainland region on Earth where the number of naturalized alien species exceeds the one of native species is the Chilean province of Iquique. This stark contrast between island and mainland regions is highly significant for RRN (Mann–Whitney *U* test: *P* < 0.001), and even more so for RRI (Fig. 1B). Islands have a median of 2.4 invasive species per 100 native species, with nine islands harbouring even >25 invasive species per 100 native species. Mainland regions, in contrast, have a median of only 0.7 invasive species per 100 native species, with a maximum of seven in Shanghai. Again, this difference in RRI between islands and mainland regions is highly significant (Mann–Whitney *U* test: *P* < 0.001). The percentage of naturalized species that have become invasive, in contrast, does not differ significantly between island and mainland regions (median for islands: 11.2 %; median for mainland regions: 9.2 %, Mann–Whitney *U* test: *P* = 0.35) **[see****Supporting Information—Fig. S2****]**. This implies that the substantial differences in RRI do not stem from higher probabilities of naturalized species to become invasive on islands. Thus, at the global scale the probability of a naturalized species becoming invasive is similar on islands and in mainland regions.

### The drivers of the relative richness of naturalized and invasive plants

**Figure 2.**
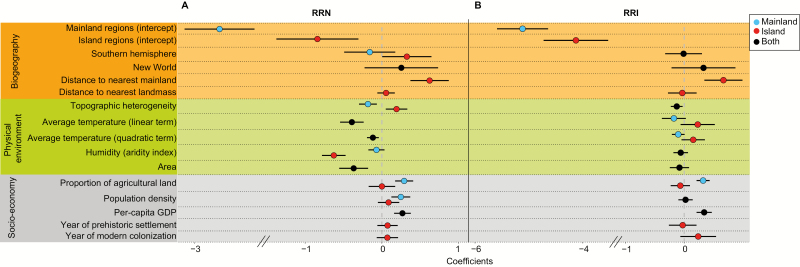
Regression coefficients of the 14 predictor variables that explain the relative richness of naturalized (RRN) (A) and invasive (RRI) (B) plants relative to native plants of 761 and 359 regions worldwide, respectively. Data on human colonization were only available for islands. The coefficients were estimated by GLMMs, and the lines show their 95 % confidence intervals. Separate coefficients for mainland vs. island regions were used only if this separation yielded a lower AIC value than a single coefficient for both region kinds. Numerical predictor variables were standardized. The full models are provided in Table 1.

**Figure 3.**
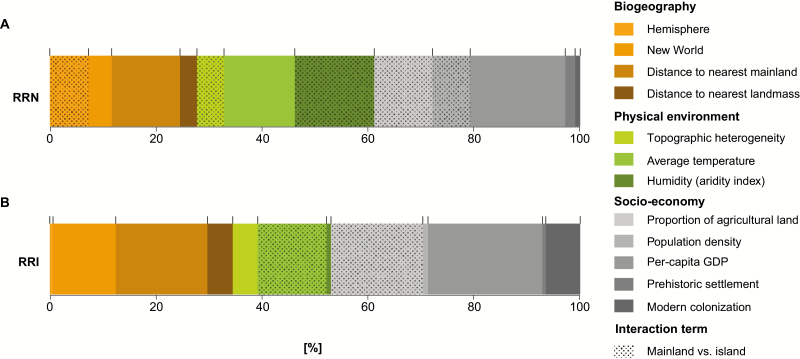
Relative importance of biogeographic (orange), physical environment (green) and socio-economic (grey) variables for explaining the relative richness of naturalized (RRN) (A) and invasive (RRI) (B) plants worldwide. Mainland vs. island region and region area are core predictors included in all predictor subsets fitted for this analysis, and therefore no assessment of relative importance applies to them. For relative importance of variables for explaining absolute naturalized and invasive species numbers, **see****Supporting Information—Fig. S4**.

Our regression models show that the global RRN is determined by an intricate mix of biogeographical, environmental and socio-economic variables (Fig. 2). Current values of socio-economic factors (per-capita GDP, population density, proportion of agricultural land) that reflect the intensity of anthropogenic disturbances and propagule pressure (Pyšek *et al.* 2010; Essl *et al.* 2011) are particularly strong predictors of RRN (Fig. 3A). With regard to biogeographical factors, we find a strong island–mainland difference, with islands in the southern hemisphere having higher RRN than those in the northern hemisphere. Conversely, for mainland regions, there is no difference between hemispheres, and there is also no significant difference in RRN between the Old and New World. With respect to climate, the relationship of RRN to mean temperature is unimodal with the highest values of RRN found in regions in the mid-temperature range (e.g. Mediterranean and warm-temperate regions). Furthermore, although humid islands have lower RRN than more arid islands, there was no corresponding effect for mainland regions (Table 1A). The importance of other factors in explaining RRN also differs substantially between island and mainland regions. Greater topographic heterogeneity (measured as the standard deviation of elevation within a region) is related to higher RRN on islands, but to lower RRN in mainland regions. This indicates that mountainous regions on mainlands harbour relatively fewer naturalized plants than lowlands, while this is not the case for islands. For islands, increasing distance to the nearest continent increases RRN, while year of pre-historic and of modern colonization show no effect.

The RRI also shows substantial differences between mainland and island regions, but these patterns are partly driven by other factors than in the case of RRN (Fig. 2). For RRI of both mainland regions and islands, indicators of human pressure (per-capita GDP and proportion of agricultural land) are highly important predictors (Fig. 3B). Moreover, RRI increases with increasing proportion of agricultural land in mainland regions, but not on islands (Table 1B). Higher topographic heterogeneity decreases RRI, both in mainland regions and on islands, indicating that mountains—which are less modified by humans—have lower proportions of invasive alien species. In contrast to plant naturalizations, average annual temperature has a marginal effect on RRI on both island and mainland regions. Isolated (sub)tropical islands show the highest RRI of all regions as exemplified by Hawai’i, Mauritius and the Seychelles.

## Discussion

Our analysis yields the first global-scale insights into the patterns of the relative richness of naturalized and invasive plant species compared to native species richness and the underlying factors. We found that both RRN and RRI are substantially higher on islands compared to mainland regions, and that on more than a quarter of the islands, naturalized alien species numbers already exceed the size of the native species pool. This pattern in relative richness of alien plant species is distinct from the one of absolute alien plant species richness (van Kleunen *et al.* 2015; Dawson *et al.* 2017; Moser *et al.* 2018) (Supporting Information—Table S2). In particular, while Dawson *et al.* (2017) had already shown that islands are hotspots of global naturalized species richness across eight taxonomic groups (including vascular plants), we for the first time show the extent of this difference, its prevalence across invasion stages, and provide an in-depth analysis of the underlying drivers.

The two metrics we have used in the analyses, RRN and RRI, are sensitive to the size of both the native and alien species pools, which allows us to assess the relative consequences of plant invasions on floristic composition and impacts. Socio-economic pressures are more important than biogeography and the physical environment for both RRN and RRI, but the relative importance of socio-economic factors is higher when explaining RRI (~50 % of the explained variation) than RRN (~40 % of the explained variation; Fig. 3; **see****Supporting Information—Fig. S3**). This finding challenges the expectation of Williamson (2006) that socio-economic pressures are more relevant for plant naturalization than for invasive plants. Our result shows that human pressures such as the level of economic prosperity (per-capita GDP), land-use intensity (proportion of agricultural land) and human population density are pivotal for explaining plant invasions on a global scale. These socio-economic variables are proxies for the relevant causes of biological invasions such as propagule pressure (Pyšek *et al.* 2010; Dyer *et al.* 2017), land use and associated habitat destruction (Chytrý *et al.* 2008a) and eutrophication (Scherer-Lorenzen *et al.* 2007), i.e. factors that facilitate plant naturalization, and even more profoundly increase the relative richness of invasive plants. As a future challenge, there is a critical need for identifying the actual socio-economic pressures that facilitate invasions, and to improve our understanding of their importance for different steps in the invasion process.

Biogeography also explains a significant amount of variation in the relative richness of naturalized and invasive plants, with the island–mainland differentiation being by far the most important factor included in our analysis. However, the importance of explanatory variables differs between RRN and RRI, and also between mainland and island regions. Islands harbour disproportionally more alien plants with deleterious impacts on the environment and which ultimately pose a major threat to native species survival (Sax and Gaines 2008; McGeoch *et al.* 2010). Island floras are thus not only hotspots of anthropogenic mixing and subsequent homogenization of regional species pools (Winter *et al.* 2010; Rosenblad and Sax 2017), but also hotspots of alien species with negative environmental impacts (i.e. invasive aliens). While it has been repeatedly shown that islands are more affected by naturalized species (e.g. Vitousek 1988; Vitousek *et al.* 1997; Lonsdale 1999; Kueffer *et al.* 2010 for plants; Dyer *et al.* 2017 for birds; Capinha *et al.* 2017 for amphibians and reptiles; Dawson *et al.* 2017 for several taxonomic groups) than mainland regions, the precise nature and extent of these differences on a global scale for vascular plants was unknown, nor had it been tested whether islands and mainland regions differ in invasive alien species richness. We find that, on average, islands harbour ~6-fold relative richness of naturalized species, and >3-fold relative richness of invasive species compared to mainland regions. With more than a quarter of all islands in our analysis having accumulated more naturalized alien plant species than are native to the same island, we find that the magnitude of anthropogenic permanent additions to the regional species pool—and the relative richness of alien species with environmental impacts—have become pervasive for many islands.

The higher RRN and RRI on islands compared to mainland regions may be influenced by the smaller size and peculiarity (e.g. reduced taxonomic and functional diversity; Whittaker *et al.* 2017) of the native plant species pool on islands compared to mainland regions of equal size (Vitousek *et al.* 1997; Lonsdale 1999; Weigelt *et al.* 2016; Moser *et al.* 2018), and partly also by high colonization pressure. For instance, the scarcity on islands of native plant species for utilitarian or ornamental purposes was a likely cause of particularly high numbers of alien plants being introduced for cultivation on islands. Further, islands are characterized by high levels of imports of goods (including within-country imports for islands that belong to mainland countries), which further increases propagule pressure in particular for hitchhiker species. It has been recently demonstrated that colonization pressure is essential to understanding global alien bird species richness (Dyer *et al.* 2016). However, disentangling the relative contribution of elevated colonization pressure and smaller native species numbers on islands for plants will require further data (e.g. numbers of introduced plants, and import volumes of goods, such as live plants, seeds and soil, that are known to bear high risks of introducing propagules) that could serve as proxies of the current, and ideally also historic, colonization pressure.

We identified geographic isolation of islands as a key factor for RRN and RRI alike—corroborating a recent finding for naturalized species numbers on (sub)tropical islands for several taxonomic groups (Moser *et al.* 2018). Thus, RRN and RRI are highest in regions that are far away from major world economies. Island isolation—as measured by increasing distance from the nearest continent—promotes plant invasions, most likely because colonization by natural means is more difficult for distant islands (Weigelt and Kreft 2013); this, in turn, leads to fewer native species and more unoccupied niches (Triantis *et al.* 2012; Whittaker *et al.* 2017; Moser *et al.* 2018). Alarmingly, remote islands are not only most affected by plant invasions, they also harbour particularly high numbers of endemic species (Weigelt *et al.* 2016) that are put at risk from invasive plants. High rates of plant naturalization on islands have prevailed since the 19th century, and there are no signs of a slow-down in naturalized plant accumulation rates (Seebens *et al.* 2017, 2018).

Interestingly, year of pre-historic and modern colonization show no effect on RRN on islands at a global scale. This lack of legacies of historic human colonization on current plant naturalization is surprising, because several studies have shown that long-term effects of historic socio-economic developments may be detectable for at least a century (Essl *et al.* 2011), and that they are particularly pronounced for invasive plants (Rouget *et al.* 2016). Most likely, more recent and pervasive changes in socio-economic pressures mask any potential signatures of early human colonization of islands on current RRN and RRI.

We found no significant differences in RRN and RRI between the Old and New World, adding a new perspective to the expectation of di Castri (1989) that Europe is predominantly an exporter of alien species, at least if native species richness is considered (Lonsdale 1999). At least in the recent decades, Europe has been a net importer of alien plant species (Seebens *et al.* 2015). The expectation that regions in the southern hemisphere are more invaded than those in the northern hemisphere could only be confirmed for RRN on islands, but not for mainland regions, and not at all for RRI. This result is consistent with the finding of van Kleunen *et al.* (2015) that Pacific islands—which are mostly located on the southern hemisphere—have received many more naturalized plants than they have exported to other regions.

The relative importance of physical environmental factors declined from ~30 % of the explained variation for RRN to ~20 % of the explained variation for RRI (Fig. 3). Warm-temperate and sub(tropical) regions exhibit the highest RRN compared to regions in the tropics or in cooler regions, whereas temperature has a marginal effect on the RRI on islands and in mainland regions. In addition, humidity plays an important role in explaining RRN on islands. This indicates that climate is an important filter for the relative richness of alien plants, in particular for naturalized species.

Topographical heterogeneity has a substantial, though divergent influence on RRN on island and mainland regions. The fact that mountainous mainland regions are less affected by plant naturalizations (Alexander *et al.* 2011) than their island counterparts (Kueffer *et al.* 2010) may indicate that topographically heterogeneous islands offer unoccupied niche space to newcomers that is not, or to a lesser degree, available in mainland regions with their longer evolutionary histories. In addition, mountains on islands often are less isolated from densely populated regions (e.g. at the coasts) than mainland mountains. However, RRI is negatively affected by topographic heterogeneity both on islands and in mainland regions indicating that invasive species currently are less common in mountains than in lowland regions (Pauchard *et al.* 2015). As alien species are predominantly introduced into more densely populated lowland areas, from where they spread into mountains (Alexander *et al.* 2011), this negative relationship may partly be caused by propagule pressure and historical legacies, i.e. the time needed to spread from lowland areas into mountains, and to become invasive.

While our analyses are based on a unique and robust data set, they also have specific limitations that warrant discussion. Since we lack explicit data on propagule pressure and colonization pressure for plants, we use proxy variables (e.g. human population density, per-capita GDP). Propagule pressure and colonization pressure are known to be key factors for explaining the success of alien plants and should thus be taken explicitly into account to disentangle region invasibility and invasion success as measured with the metrics we have used here (Lonsdale 1999; Diez *et al.* 2009; Richardson and Pyšek 2012). However, data that allow direct quantification of propagule pressure (e.g. numbers of introduced propagules or plants, number of introduction events) and colonization pressure (number of introduced species) are not available for plants on a global scale, and even largely absent for smaller regions. Thus, using proxy variables for propagule and colonization pressures is currently the best possible approach. Further, the addition of explanatory variables that might allow to capture currently under-investigated potential drivers of global plant invasions (e.g. improved measures of land-use intensity and colonization pressure) might allow for more nuanced insights into invasions; however, gaps and biases in available data have to be overcome to make it possible. Finally, sampling intensity varies between regions and influences recorded numbers of naturalized and invasive plants. While such variation in sampling intensity cannot be completely excluded, the GloNAF database is by far the most complete source using a wide range of data sources and following standardized criteria (van Kleunen *et al.* 2015; Pyšek *et al.* 2017).

## Conclusions

Of the five hypotheses that have been proposed to explain the geographic patterns of alien species richness and which we tested with our data set, we could clearly confirm the mainland–island differentiation. We did not find support for the expectation that socio-economic pressures are more important for naturalized than invasive species (Williamson 2006), as we found that socio-economic pressures explain more of the variation in RRI than in RRN. The expectation that regions in the southern hemisphere are more invaded could only be confirmed for RRN on islands, and the Old vs. New World dichotomy could not be confirmed at all. We found no evidence that time elapsed since colonization of islands influences the current RRN or RRI. Further, the percentages of naturalized species that have become invasive (median for islands: 11.2 %; median for mainland regions: 9.2 %) support one of the oldest and most widely cited ‘rules of thumb’ in invasion ecology, i.e. the tens-rule (Williamson and Fitter 1996) for plants.

Our results have three major implications for management and policy. First, given the high relative importance of socio-economic pressures for RRI, regions facing high human pressures like habitat degradation and intensive land use provide more opportunities for naturalized species to spread and cause negative impacts on the environment. Given the considerable recent increases in these pressures (Waters *et al.* 2016), the emergence of new pressures, such as climate change (Walther *et al.* 2009; Dullinger *et al.* 2017), and the fact that time lags of several decades are frequent in large-scale plant invasions responding to these pressures (Seebens *et al.* 2015), a further increase in the RRI is inevitable unless effective management policies are put in place (Tittensor *et al.* 2014). Second, alien species are already implicated in the majority of extinctions of species on islands (Sax and Gaines 2008). Our results support recent findings (Moser *et al.* 2018) that the future of extant island biodiversity may become even more severely compromised by biological invasions. Third, to meet international biodiversity goals such as the Aichi biodiversity targets 9 (on reducing the impacts of invasive species) and 12 (on preventing extinctions) (McGeoch *et al.* 2016), it will be pivotal to disrupt the connection between socio-economic development (e.g. per-capita GDP, trade; Pyšek *et al.* 2010; Seebens *et al.* 2015) and rising levels of naturalized and invasive plants by implementing comprehensive and effective biosecurity policies targeted to pathway management, early detection and rapid response (Early *et al.* 2016), especially focussing on islands. Finally, we believe that for informed invasive alien species policies and management it is important to take into account insights from the analyses of the relative and absolute richness of alien species.

## Supporting Information

The following additional information is available in the online version of this article— 

**Figure S1.** The global richness of native and alien vascular plants. Shown are the numbers of native (A), naturalized (B) and invasive (C) plant species. Boxplots show numbers of native, naturalized and invasive plant species for island and mainland regions.

**Figure S2.** The correlation of the relative richness of naturalized (A) and invasive (B) plants, i.e. number of naturalized and invasive plant species relative to native plant species numbers, and correlation of invasive per naturalized species numbers (C). Relative richness of naturalized and invasive plants are significantly different between islands and mainland regions (A, B; Mann-Whitney *U* test: *p* < 0.001 in both cases), but there is no such difference between the ratio of invasive per naturalized species numbers (C; Mann-Whitney *U* test: *p* = 0.35).

**Figure S3.** Partitioning of the explained variation in regression models of global alien vascular plant species numbers grouped into biogeographic, physical environment and socio-economic variables. Shown are the ratios of naturalized (A) and invasive (B) plant species per native ones, and the absolute numbers of naturalized (C) and invasive (D) plant species. Biogeographic variables include hemisphere, Old vs. New World and the island-specific variables distance to nearest continental mainland/landmass; physical environment variables include topographic heterogeneity, temperature and humidity; and socio-economic variables include human population density, per-capita GDP, proportion of agricultural land, and human colonization histories on islands (Table S1). Intersections of circles in the diagrams represent the variation jointly explained by two or three classes of variables; negative values may occur due to suppression effects and are not shown. Mainland vs. island region and region area were core predictors included in all predictor subsets fitted for this analysis; these predictors explain variation in the models not shown here.

**Figure S4**. Relative importance of biogeographic (orange), physical environment (green), and socio-economic (grey) variables for explaining absolute numbers of naturalized (A) and invasive (B) species per region. Mainland vs. island region and region area were core predictors included in all predictor subsets fitted for this analysis, and therefore no assessment of relative importance applies to them.

**Table S1.** Explanatory variables used in the regression models, the underlying rationale, the main data sources used, and the grouping into broader classes (biogeography, physical environment, socio-economy).

**Table S2.** The generalized linear mixed effects models (GLMMs) of absolute numbers of naturalized (A) and invasive (B) plant species per region world-wide. GLMMs use a Poisson-distribution as response and a total of 14 predictor variables. Note that data on human colonization were only available for islands. Predictors were assessed for significantly different effects on mainland vs. island regions by means of interactions with this binary factor, and subjecting these interactions to a backward model search based on lower AIC; for the retained terms each of the two separate coefficients for mainland and island regions states the predictor effect at an absolute scale. Random effect intercept terms with sovereign state, TDWG continent and zonobiome as (orthogonal) grouping factors acknowledge for political/socio-economic, biogeographic, spatial and climatic correlations among regions, with an additional observation-level random effect term accounting for Poisson-distribution overdispersion. Numerical predictor variables were standardized. Estimated standard deviations of random effects: A: sovereign states: 0.38, TDWG continent: 0.58, zonobiome: 0.31, observation-level: 0.70; B: sovereign states: 0.68, TDWG continent: 0.62, zonobiome: 0.36, observation-level: 0.44.

**Table S3.** List of regions (n = 838) included in the analyses. Given are ISO country code a region belongs to, region name, the analysis a region was included in, numbers of native, naturalized and invasive plant species, and the explanatory variables used for analyses.

plz051_suppl_Supplementary_Appendix_S1

plz051_suppl_Supplementary_Tables

## Sources of Funding

This study was supported by grants from the Deutsche Forschungsgemeinschaft (DFG, grant KL 1866/9-1 to M.v.K. and W.D., grant 264740629 to M.v.K., grant SE 1891/2-1 to H.S. and grant FZT 118 to iDiv supporting M.W.), the FWF (grants I3757-B29 and I2086-B16 to F.E., D.M., T.M., S.D., H.S., B.L.). P.P. and J.P. were supported by EXPRO grant no. 19-28807X (Czech Science Foundation), and long-term research development project RVO 67985939 (the Czech Academy of Sciences).

## Contributions by the Authors

M.v.K., P.P., W.D., F.E., J.P., E.W., M.W., H.K., P.W. are the core GloNAF project members, which searched for and coordinated the collection of inventories of naturalized and invasive alien plants, D.M. and B.L. searched and extracted the environmental and socio-economic variables, and all other authors contributed naturalized plants inventories or other data. T.M. led the analyses, F.E. the writing, with major inputs from all GloNAF core team members and from S.D., T.M, H.S. and B.L., and further inputs from all other authors.

## Conflict of Interest

None declared.
