# Supplementary material for: Drivers of the relative richness of naturalized and invasive plant species on Earth
Source: AoB Plants. 2019 Sep 4;11(5):plz051. doi: 10.1093/aobpla/plz051 (PMC6795282; doi:10.1093/aobpla/plz051)
Supplement: plz051_suppl_Supplementary_Appendix_S1 [file plz051_suppl_supplementary_appendix_s1.docx]

Drivers of the relative richness of naturalized and invasive plant species on Earth

Franz Essl^a^, Wayne Dawson^b^, Holger Kreft^c^, Jan Pergl^d^, Petr Pyšek^d,e,f^, Mark van Kleunen^g,h^, Patrick Weigelt^c^, Thomas Mang^a^, Stefan Dullinger^a^, Bernd Lenzner^a^, Dietmar Moser^a^, Noëlie Maurel^g^, Hanno Seebens^i^, Anke Stein^g^, Ewald Weber^j^, Cyrille Chatelain^k^, Inderjit^l^, Piero Genovesi^m,n^, John Kartesz^o^, Olga Morozova^p^, Misako Nishino^o^, Pauline M. Nowak^q^, Shyama Pagad^r^, Wen-sheng Shu^s^, Marten Winter^t^

^a^Division of Conservation, Landscape and Vegetation Ecology, Department of Botany and Biodiversity Research, University of Vienna, Rennweg 14, 1030 Vienna, Austria.

^b^Department of Biosciences, Durham University, South Road, Durham, DH1 3LE, United Kingdom.

^c^Biodiversity, Macroecology and Biogeography, University of Goettingen, Büsgenweg 1, D-37077 Göttingen, Germany.

^d^The Czech Academy of Sciences, Institute of Botany, Department of Invasion Ecology, CZ-252 43 Průhonice, Czech Republic.

^e^Department of Ecology, Faculty of Science, Charles University, Viničná 7, CZ-128 44 Prague, Czech Republic.

^f^Centre for Invasion Biology, Department of Botany and Zoology, Stellenbosch University, Matieland 7602, South Africa.

^g^Ecology, University of Konstanz, Universitätsstrasse 10, 78457 Konstanz, Germany.

^h^Zhejiang Provincial Key Laboratory of Plant Evolutionary Ecology and Conservation, Taizhou University, Taizhou 318000, China.

^i^Senckenberg Biodiversity and Climate Research Centre (BiK-F), Senckenberganlage 25, 60325 Frankfurt am Main, Germany.

^j^Institute of Biochemistry and Biology, University of Potsdam, Maulbeerallee 1, 14469 Potsdam, Germany.

^k^Conservatoire et Jardin Botaniques de la Ville de Genève, Genève, Switzerland.

^l^Department of Environmental Studies and Centre for Environmental Management of Degraded Ecosystems, University of Delhi, Delhi 110007, India.

^m^Institute for Environmental Protection and Research (ISPRA), Via Vitaliano Brancati 48, 00144 Rome, Italy.

^n^Chair IUCN Species Survival Commission’s Invasive Species Specialist Group (ISSG), Rome, Italy.

^o^Biota of North America Program (BONAP), Chapel Hill, North Carolina 27516, USA.

^p^Institute of Geography RAS, Staromonetny, 29, 119017 Moscow, Russia.

^q^Department of Geography, University Marburg, Deutschhausstraße 10, D-35032 Marburg, Germany.

^r^IUCN Species Survival Commission’s Invasive Species Specialist Group (ISSG), University of Auckland, New Zealand.

^s^State Key Laboratory of Biocontrol and Guangdong Key Laboratory of Plant Resources, College of Ecology and Evolution, Sun Yat-sen University, Guangzhou 510275, China.

^t^German Centre for Integrative Biodiversity Research (iDiv) Halle-Jena-Leipzig, Deutscher Platz 5e, 04103 Leipzig, Germany.

*To whom correspondence should be addressed. E-mail: franz.essl@univie.ac.at. Tel.: +43 1 4277 54372. Fax: +43 1 4029675 10

# **Appendix S1**

**Supporting Figures**

**Fig. S1**


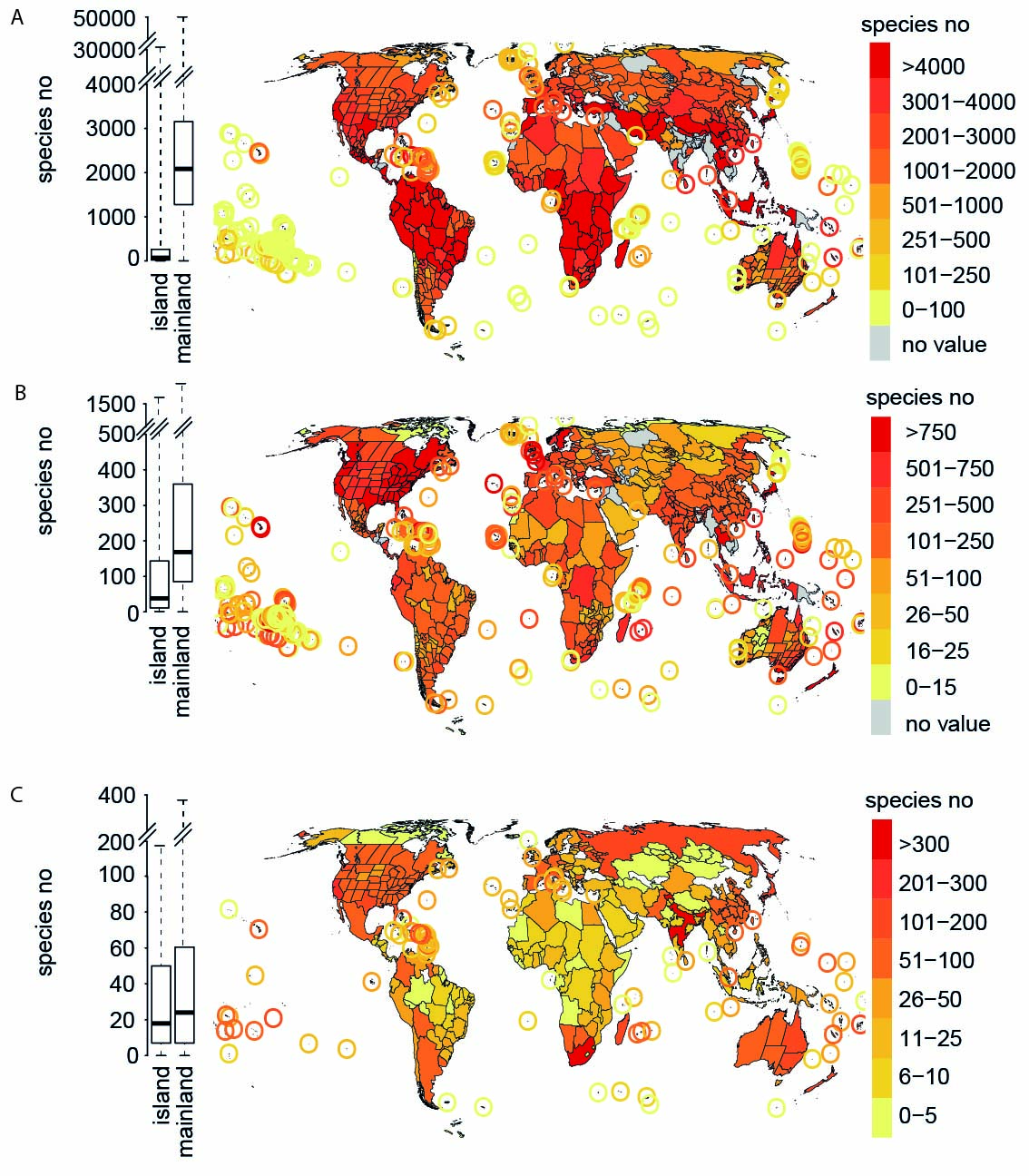


Figure S1: The global richness of native and alien vascular plants. Shown are the numbers of native (A), naturalized (B) and invasive (C) plant species. Boxplots show numbers of native, naturalized and invasive plant species for island and mainland regions.

**Fig. S2**

**
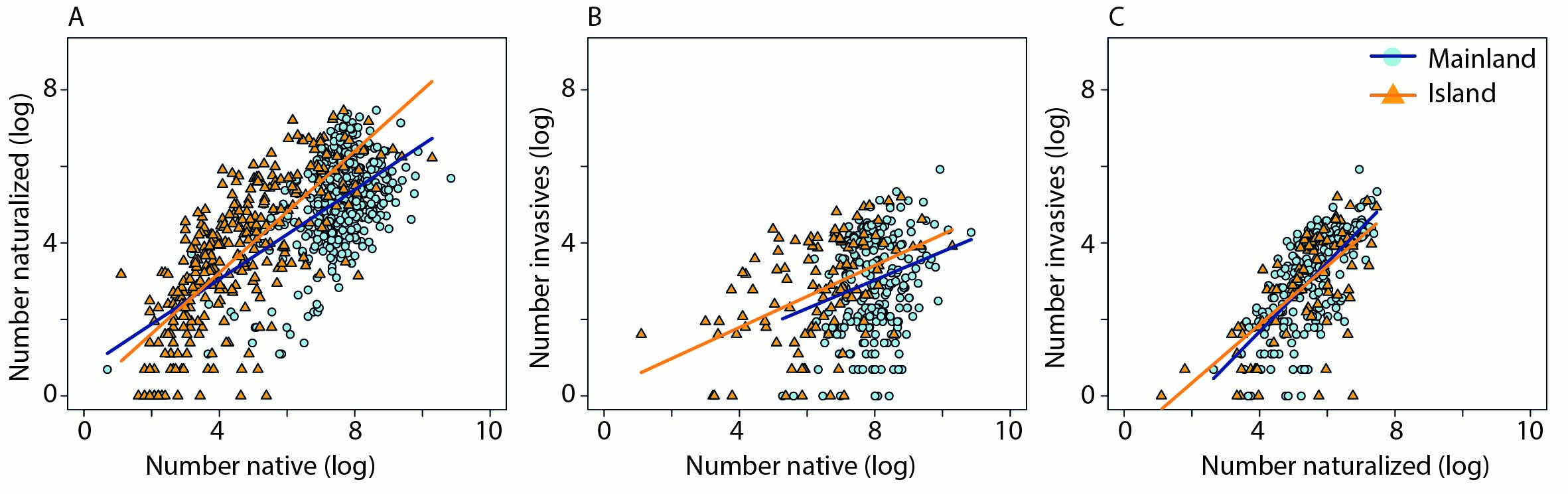
**

Figure S2: The correlation of the relative richness of naturalized (A) and invasive (B) plants, i.e. number of naturalized and invasive plant species relative to native plant species numbers, and correlation of invasive per naturalized species numbers (C). Relative richness of naturalized and invasive plants are significantly different between islands and mainland regions (A, B; Mann-Whitney *U* test: *p* < 0.001 in both cases), but there is no such difference between the ratio of invasive per naturalized species numbers (C; Mann-Whitney *U* test: *p* = 0.35).

**Fig. S3**


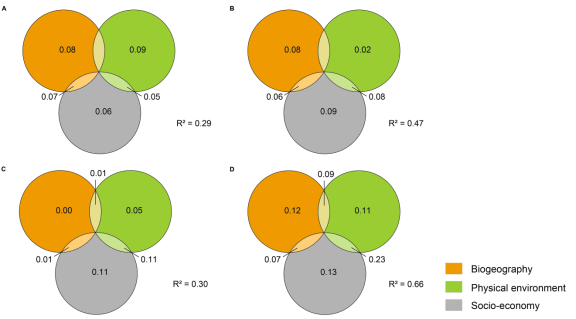


Figure S3: Partitioning of the explained variation in regression models of global alien vascular plant species numbers grouped into biogeographic, physical environment and socio-economic variables. Shown are the ratios of naturalized (A) and invasive (B) plant species per native ones, and the absolute numbers of naturalized (C) and invasive (D) plant species. Biogeographic variables include hemisphere, Old vs. New World and the island-specific variables distance to nearest continental mainland/landmass; physical environment variables include topographic heterogeneity, temperature and humidity; and socio-economic variables include human population density, per-capita GDP, proportion of agricultural land, and human colonization histories on islands (Table S1). Intersections of circles in the diagrams represent the variation jointly explained by two or three classes of variables; negative values may occur due to suppression effects and are not shown. Mainland vs. island region and region area were core predictors included in all predictor subsets fitted for this analysis; these predictors explain variation in the models not shown here.

Fig. S4


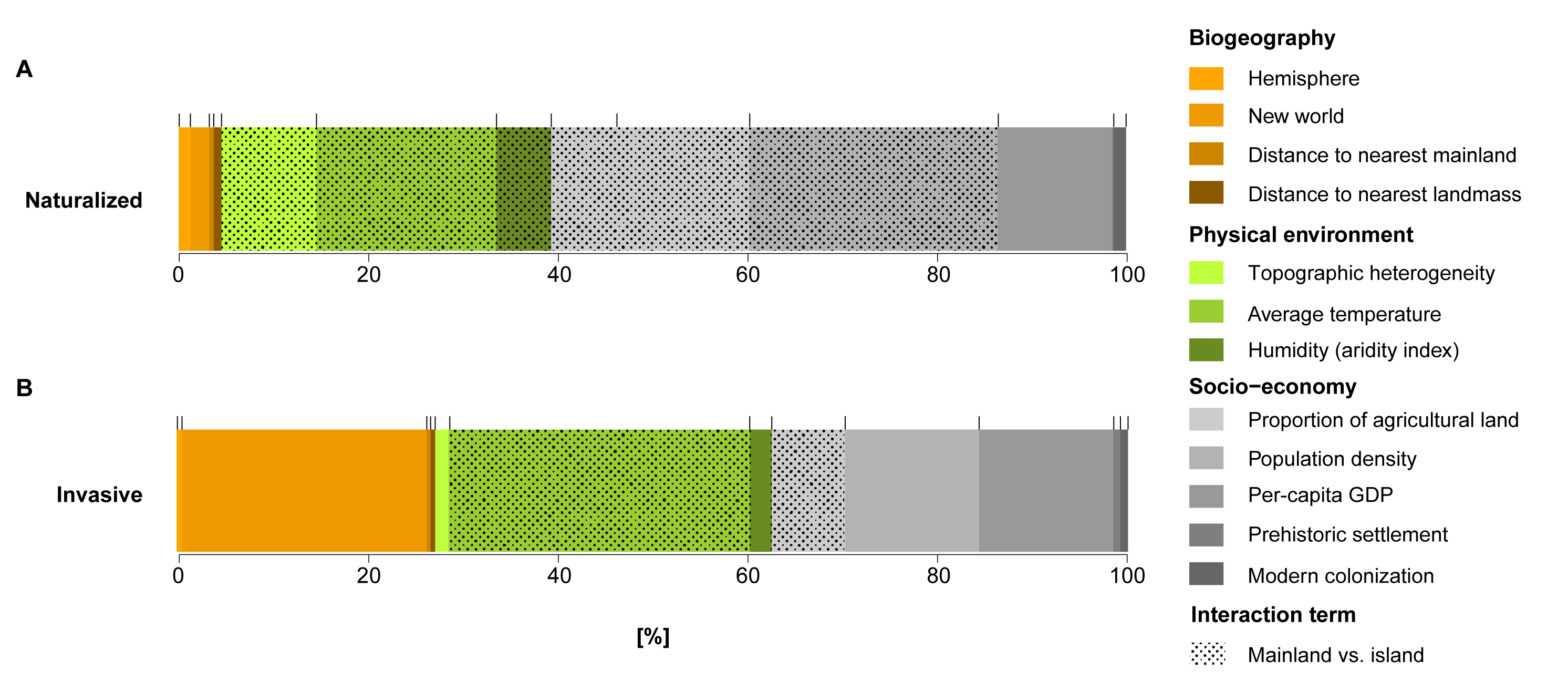


Figure S4: Relative importance of biogeographic (orange), physical environment (green), and socio-economic (grey) variables for explaining absolute numbers of naturalized (A) and invasive (B) species per region. Mainland vs. island region and region area were core predictors included in all predictor subsets fitted for this analysis, and therefore no assessment of relative importance applies to them.

**Supporting Tables**

Table S1: Explanatory variables used in the regression models, the underlying rationale, the main data sources used, and the grouping into broader classes (biogeography, physical environment, socio-economy).

| **Explanatory variables** | **Main data sources** | **Rationale** | **Domain** |
| --- | --- | --- | --- |
| Hemisphere | Own calculation (Essl et al. ined.) | Regions in the southern hemisphere have distinct, but isolated native floras that often lack co-evolutionary experience with agricultural societies | Biogeography |
| Old vs. New World | Own calculation (Essl et al. ined.) | Regions in the Old World often have long histories of colonization and agriculture, while European settlers may have brought plants into the New World | Biogeography |
| Distance to nearest continental mainland (islands only) | Weigelt & Kreft (2013) | Increasing island isolation decreases natural colonization rates and thus may result in larger available niche space | Biogeography |
| Distance to nearest landmass (only landmasses that are larger than the focal island have been considered) (islands only) | Weigelt & Kreft (2013) | Increasing island isolation decreases natural colonization rates and thus may result in larger available niche space | Biogeography |
| Topographic heterogeneity | Digital Elevation Model, WorldClim (<http://www.worldclim.org/>), own calculation (Essl et al. ined.) | Topographic heterogeneity within a region is associated with niche availability that may affect plant invasions | Physical environment |
| Average annual temperature | WorldClim v2 for the period 1971-2000 (http://www.worldclim.org/) | Regions characterized by different average annual temperatures may provide different opportunities for plant invasions | Physical environment |
| Humidity | Own calculations (based on data from WorldClim v2 for the period 1971-2000 (http://www.worldclim.org/) | Regions of different humidity may offer different opportunities for plant invasions | Physical environment |
| Area | Own calculation (Essl et al. ined.) | Regions of different sizes may offer different opportunities for plant invasions | Biogeography |
| Human population density | HYDE-database (Klein Goldewijk et al. 2011); data for the year 2010 | Population density is an important predictor for ecosystem degradation, a factor known to promote plant invasions | Socio-economy |
| Per-capita GDP | Gennaioli et al. (2014), United Nations Statistics Division (2015), CIA (2013); data for the year 2010 | Per-capita GDP is an important predictor of socio-economic activities including trade, a factor known to promote plant invasions | Socio-economy |
| Proportion of agricultural land | HYDE-database (Klein Goldewijk et al. 2011); data on arable land for the year 2000 | The proportion of agricultural land is a proxy for anthropogenic habitat destruction, a factor known to promote plant invasions | Socio-economy |
| Year of first pre-historic settlement (islands only) | Own data (Essl et al. ined.), different data sources | Longer pre-historic (i.e. colonization by people living in pre-industrial societies) settlement history may result in a longer history of habitat degradation and introduction of plants | Socio-economy |
| Year of modern colonization (islands only) | Own data (Essl et al. ined.), different data sources | Modern (European) colonization marks the onset of advanced agricultural and industrial societies and global trade associated with high rates of habitat degradation and plant introductions | Socio-economy |

Table S2: The generalized linear mixed effects models (GLMMs) of absolute numbers of naturalized (A) and invasive (B) plant species per region world-wide. GLMMs use a Poisson-distribution as response and a total of 14 predictor variables. Note that data on human colonization were only available for islands. Predictors were assessed for significantly different effects on mainland vs. island regions by means of interactions with this binary factor, and subjecting these interactions to a backward model search based on lower AIC; for the retained terms each of the two separate coefficients for mainland and island regions states the predictor effect at an absolute scale. Random effect intercept terms with sovereign state, TDWG continent and zonobiome as (orthogonal) grouping factors acknowledge for political/socio-economic, biogeographic, spatial and climatic correlations among regions, with an additional observation-level random effect term accounting for Poisson-distribution overdispersion. Numerical predictor variables were standardized. Estimated standard deviations of random effects: A: sovereign states: 0.38, TDWG continent: 0.58, zonobiome: 0.31, observation-level: 0.70; B: sovereign states: 0.68, TDWG continent: 0.62, zonobiome: 0.36, observation-level: 0.44.

| **Predictor** | **Mainland vs. island regions** | **Coefficient** | **Std. error** | ***p*-value** |
| --- | --- | --- | --- | --- |
| Intercept - mainland regions | mainl | 3.99 | 0.31 | < 0.001 |
| Intercept - island regions | isl | 5.16 | 0.34 | < 0.001 |
| *Biogeographic variables* |  |  |  |  |
| Southern hemisphere | mainl & isl | 0.09 | 0.13 | 0.50 |
| New World | mainl & isl | 0.12 | 0.33 | 0.71 |
| Distance to nearest mainland | isl | 0.08 | 0.13 | 0.50 |
| Distance to nearest landmass | isl | 0.00 | 0.06 | 0.96 |
| *Physical environment variables* |  |  |  |  |
| Topographic heterogeneity | mainl | 0.22 | 0.06 | < 0.001 |
|  | isl | 0.44 | 0.07 | < 0.001 |
| Average annual temperature (linear term) | mainl | -0.26 | 0.09 | < 0.01 |
|  | isl | -0.11 | 0.13 | 0.42 |
| Average annual temperature (quadratic term) | mainl | -0.19 | 0.05 | < 0.001 |
|  | isl | -0.32 | 0.08 | < 0.001 |
| Humidity (aridity index) | mainl | 0.22 | 0.06 | < 0.001 |
|  | isl | -0.02 | 0.08 | 0.77 |
| Area | mainl & isl | 0.96 | 0.09 | < 0.001 |
| *Socio-economic variables* |  |  |  |  |
| Proportion of agricultural land | mainl | 0.38 | 0.06 | < 0.001 |
|  | isl | 0.14 | 0.09 | 0.13 |
| Population density | mainl | 0.54 | 0.07 | < 0.001 |
|  | isl | 0.11 | 0.08 | 0.15 |
| Per-capita GDP | mainl & isl | 0.38 | 0.06 | < 0.001 |
| Year of pre-historic settlement | isl | -0.01 | 0.07 | 0.93 |
| Year of modern colonization | isl | 0.09 | 0.07 | 0.21 |

B

| **Predictor** | **Mainland vs. island regions** | **Coefficient** | **Std. error** | ***p*-value** |
| --- | --- | --- | --- | --- |
| Intercept - mainland regions | mainl | 2.58 | 0.34 | < 0.001 |
| Intercept - island regions | isl | 3.01 | 0.39 | < 0.001 |
| *Biogeographic variables* |  |  |  |  |
| Southern hemisphere | mainl & isl | 0.04 | 0.18 | 0.80 |
| New World | mainl & isl | 0.94 | 0.41 | 0.02 |
| Distance to nearest mainland | isl | 0.11 | 0.18 | 0.55 |
| Distance to nearest landmass | isl | -0.18 | 0.14 | 0.19 |
| *Physical environment variables* |  |  |  |  |
| Topographic heterogeneity | mainl & isl | 0.11 | 0.05 | 0.05 |
| Average annual temperature (linear term) | mainl | -0.14 | 0.12 | 0.24 |
|  | isl | 0.48 | 0.17 | < 0.01 |
| Average annual temperature (quadratic term) | mainl | -0.31 | 0.06 | < 0.001 |
|  | isl | -0.16 | 0.12 | 0.21 |
| Humidity (aridity index) | mainl & isl | 0.16 | 0.07 | 0.02 |
| Area | mainl & isl | 0.93 | 0.08 | < 0.001 |
| *Socio-economic variables* |  |  |  |  |
| Proportion of agricultural land | mainl | 0.10 | 0.06 | 0.08 |
|  | isl | -0.14 | 0.09 | 0.11 |
| Population density | mainl & isl | 0.41 | 0.07 | < 0.001 |
| Per-capita GDP | mainl & isl | 0.30 | 0.07 | < 0.001 |
| Year of pre-historic settlement | isl | 0.19 | 0.14 | 0.17 |
| Year of modern colonization | isl | 0.09 | 0.13 | 0.48 |

**Table S3**

Table S3: List of regions (n = 838) included in the analyses. Given are ISO country code a region belongs to, region name, the analysis a region was included in, numbers of native, naturalized and invasive plant species, and the explanatory variables used for analyses.

This table is provided as a supplementary xlsx-file.

**Supporting References**

Gennaioli, N. et al. 2014. Growth in regions. – Journal of Economic Growth 19: 259–309.

United Nations Statistics Division 2015. Per capita GDP at current prices - US dollars. National Accounts Estimates of Main Aggregates. – <http://data.un.org/Data.aspx?q=per+capita+GDP&d=SNAAMA&f=grID%3a101%3bcurrID%3aUSD%3bpcFlag%3a1>.

CIA 2013. The World Factbook 2013-2014. – Washington, Central Intelligence Agency.
